# Temperature Changes Affect the Vulnerability of Cotton Bollworms, *Helicoverpa armigera* (Hübner)

**DOI:** 10.3390/insects17010040

**Published:** 2025-12-28

**Authors:** Jian Huang, Pengfei Wu, Wenyuan Xing, Xiaojun Wang

**Affiliations:** 1 Institute of Desert Meteorology, China Meteorological Administration, Urumqi 830002, China; wangxj@idm.cn; 2 Wulanwusu National Special Test Field for Comprehensive Meteorological Observation, Urumqi 830002, China; 3 Wulanwusu Ecology and Agrometeorology Observation and Research Station of Xinjiang, Urumqi 830002, China; 4 Urumqi Meteorological Satellite Ground Station, Urumqi 830011, China; wpf7848@sina.com (P.W.); xwy6851@sina.com (W.X.)

**Keywords:** climate warming, cotton bollworm, vulnerability

## Abstract

The cotton bollworm, *Helicoverpa armigera* (Hübner), a cosmopolitan agricultural pest, inflicts severe impacts on global agriculture. As a poikilotherm, it was highly susceptible to climate change, yet critical gaps persist in understanding how its sensitivity interacts with climatic shifts—knowledge essential for integrated pest management (IPM). We, therefore, analyzed *H. armigera*’s susceptibility to temperature variations using long-term pest population and meteorological data from Maigaiti and Bachu Counties (southern Xinjiang) and Shawan County (northern Xinjiang). The results showed *H. armigera* populations increased overall, with reduced interannual fluctuation magnitude. The main meteorological factors influencing the interannual population changes of *H. armigera* in Maigaiti, Bachu, and Shawan were *T*_max_ difference in winter (98.0%), *T*_min_ difference in May (80.7%), and *T*_min_ difference in July (99.4%), respectively. Higher winter temperature (particularly February) reduced the spring population sizes across all three regions, with only the population in Bachu showing a significant correlation. For annual populations, warmer winter caused a significant decline in Bachu, a marked increase in Maigaiti, and a non-significant rise in Shawan. Summer temperature below 33 °C boosted populations in all regions; above 33 °C, the Maigaiti population declined non-significantly, while the Bachu population dropped significantly. Climate warming advanced the pest’s first appearance, delayed its disappearance, and extended its active period, increasing population size—a trend projected to intensify in the future. Maigaiti and Shawan populations were governed by *T*_max_ in winter and *T*_min_ in July, respectively, whereas the Bachu population was constrained by temperature differences during multiple key growth and development periods throughout the year. These divergent regulatory patterns and climatic responses reflect varying vulnerability levels, providing a theoretical basis for targeted *H. armigera* control.

## Simple Summary

The cotton bollworm (*Helicoverpa armigera*), a globally distributed agricultural pest, severely damages crops and, as a poikilotherm, is highly sensitive to climate change—yet the link between its susceptibility and climatic shifts remains poorly understood, hindering integrated pest management (IPM). We analyzed long-term *H. armigera* population data and meteorological records from Maigaiti, Bachu (southern Xinjiang), and Shawan (northern Xinjiang) to clarify its response to temperature variation. The results showed *H. armigera* populations increased overall, with reduced interannual fluctuations. The main meteorological factors influencing the interannual population changes of *H. armigera* in Maigaiti, Bachu, and Shawan were *T*_max_ difference in winter, *T*_min_ difference in May, and *T*_min_ difference in July, respectively. Higher winter temperatures (especially February) lowered the spring population sizes. Warmer winters reduced Bachu’s annual population significantly, increased Maigaiti’s markedly, and raised Shawan’s non-significantly. Summer temperatures >33 °C suppressed populations, while phenological shifts boosted them. These divergent regional responses reflect varying climatic vulnerability in *H. armigera* populations, providing insights for targeted IPM.

## 1. Introduction

Insect vulnerability is defined as the inability of insects to resist or adapt to external environmental stressors at the physiological [1,2], ecological [3], or population levels [4], including climate fluctuations [5], habitat destruction [6], and chemical disturbances [7], which in turn impairs their survival and reproductive capacity or causes population decline. Insect vulnerability to climate warming manifests in two dimensions: the capacity to sustain existing populations and the ability to disperse to more suitable new environments [8].

Climate change impacts both insects and pesticides, with temperature variations exerting a significant effect on pesticides efficacy [9]. In farmlands, the release of pheromone mixtures disrupts insect mating, reducing offspring production, suppressing pest populations, and alleviating crop damage [10]. However, sex pheromones are prone to decomposition under high temperature, impairing trapping efficiency; in high-humidity environments, their transmission distance might shortens, hindering the attraction of distant *H. armigera* adults. Global warming adversely affects natural enemy insects [11], altering the population dynamics and structure of *H. armigera*. Additionally, elevated temperatures indirectly boost *H. armigera*’s resistance, with this resistance evolving even faster than the adoption of genetically modified crop protection methods [12]. Reduced pesticide efficacy at high temperatures, pesticide volatilization, enhanced *H. armigera*’s resistance, and negative impacts of high temperatures on natural enemies collectively complicated pest control. Understanding pest vulnerability to climate change thus supports IPM and the development of targeted control strategies, ultimately improving pest control efficacy.

Cotton bollworm, *Helicoverpa armigera* (Hübner) (Lepidoptera: Noctuidae), is one of the world’s most destructive agricultural insect pests in the world [13], characterized by polyphagous, high fecundity, facultative diapause, and strong mobility [14]. As a poikilotherm, *H. armigera* is highly sensitive to climate change, which drives phenological shifts and significant interannual fluctuations [15]. Previous studies have identified key factors regulating its population dynamics, temperature range of 25–30 °C maximize the intrinsic rate of increase, female fecundity, and oviposition rate [16], while parasitism in the second instar and predation in the fifth instar are primary drivers of interannual fluctuations variability [17]. The survival rate of non-parasitized, non-predated fourth-instar larvae is significantly correlated with the Wind and Rain Intensity Index [18], and pest outbreaks depend on sufficient food resources, initial insect populations, and critical meteorological regulation [19]. Meteorological factors modulate the survival, reproduction, and dispersal of *H. armigera*: 25–30 °C shortens developmental time, boosts the intrinsic rate of increase, and increases generation number, whereas extreme temperatures (>35 °C or <15 °C) inhibit population growth—low temperatures prolong the developmental and induce facultative diapause, reducing overwintering survival, while high temperature suppresses reproduction. Relative humidity of 70–85% optimizes the egg hatching rate and larval survival; humidity < 50% causes egg dehydration and larval desiccation, while humidity > 90% promotes fungal infections (e.g., *Beauveria bassiana*) and elevates natural mortality [19]. Global climate warming has extended the optimal temperature range for *H. armigera*, increased annual generations by 1–2, and improved overwintering survival. Extreme meteorological events (e.g., heat waves and post-downpour drying) indirectly favor pest outbreaks by suppressing natural enemies (e.g., *Chrysoperla sinica* and parasitic wasps) and altering host plant growth [20]. Summer–autumn hot–dry conditions further facilitate *H. armigera* reproduction, linking climate warming and greenhouse effects to pest outbreaks [21]. Climate drives *H. armigera* outbreaks through multiple pathways: within the optimal temperature range, the developmental rate rises with temperature, shortening generation time and enhancing population growth potential; humidity modulates survival, reproduction, and behavior (with the temperature–humidity index as a key predictive tool); and abnormal photoperiods disrupt life cycles, potentially triggering outbreaks [22]. Warming advances diapausing pupa eclosion, extending the overwintering pupa period and increasing first-instar larval population, which damage early-stage wheat; future asynchrony between crop and pest growth under warming may exacerbate yield losses [4]. Temperature and precipitation affect different *H. armigera* generations distinctly, while expanded non-transgenic Bt cotton cultivation increases pest populations, *T*_mean_ in June influences first-generation adults, the last occurrence date of second-generation adults affects the second generation, and third-generation adult duration regulates the third generation [23]. While climate is widely recognized as a critical driver of *H. armigera* dynamics, overwintering pupae act as the “seed bank” for subsequent-year populations, and summer reproduction determines annual population size. However, key climatic factors governing the overwintering and summer peak periods remain understudied, and trends in interannual population fluctuations are poorly understood.

The life cycle and ecological traits of *H. armigera* exhibit distinct regional adaptations between southern and northern Xinjiang. In southern Xinjiang (Maigaiti and Bachu), the species completes four generations annually. Adults occur from late April to mid-September, with peak emergence in May, July, and late August; eggs are present from late April to early October (predominantly on cotton shoots and buds) and develop in 3–4 days. Larvae—the main damaging stage—are active from mid-May to late October, undergoing six instars over 12–18 days, with successive generations overlapping from late June to August. Pupae develop in soil (5–15 cm deep): non-overwintering pupae mature in 10–12 days (mid-June to late September), while the overwintering pupal stage (the only winter-surviving life stage) persists from late October to early April. Overwintering sites include cotton field margins, cotton-corn intercropping zones, and uncultivated land adjacent to host crops, with an average minimum temperature of −8 to −12 °C (extreme lows rarely below −15 °C) during this period. In northern Xinjiang (Shawan), *H. armigera* completes three generations annually, with cooler average temperatures prolonging developmental durations slightly. Adults emerge from mid-May to mid-September, peaking in late May (overwintering generation), early July (second generation), and early August (third generation); eggs are present from late May to early October (on cotton and other host plants) and hatch in 4–5 days. Larvae are active from early June to late October, developing for 15–20 days, with overlapping generations from late June to August. Pupae burrow 5–10 cm deep in soil: non-overwintering pupae persist for 12–15 days (mid-June to late September), while overwintering pupae survive from late October to early May [24,25]. Preferred overwintering sites include cotton field ridges, fallow land, and wheat-cotton rotation field edges [25], with winter temperatures averaging −15 to −20 °C (occasional extreme lows of −25 °C). Across both regions, pupae survive winter via cold-hardiness mechanisms (e.g., cryoprotectant accumulation and metabolic depression) [25]. The adult reproductive lifespan ranges 7–9 days in southern Xinjiang (field temperatures 25–32 °C in peak growing seasons) and 6–8 days in northern Xinjiang (average 22–28 °C).

Therefore, this study aimed to address five key objectives: (1) identify the primary meteorological factors driving affecting the interannual fluctuations in *H. armigera* populations; (2) determine the overwintering meteorological factors governing spring population sizes in the subsequent year; (3) clarify the main meteorological factors regulating summer population size during the peak reproductive; (4) evaluate the impact of phenological shifts on population size; (5) assess whether regulatory patterns differ geographically distinct *H. armigera* populations.

## 2. Materials and Methods

### 2.1. Study Sites

The study was conducted in Maigaiti County (77°28′–79°05′ E, 38°25′–39°22′ N), Bachu County (77°22′–79°56′ E, 38°47′–40°17′ N), and Shawan County (84°56′–86°08′ E, 43°19′–45°55′ N) in Xinjiang. Shawan is approximately 1100 km from Bachu and 1200 km from Maigaiti. In Maigaiti County, the warmest months are July and August (summer), with maximum temperatures of 35–38 °C and a historical extreme of 42.1 °C. January (winter) is the coldest month, with minimum temperatures of −10 to −15 °C and a historical low of −22.4 °C. Winter snowfall is scarce (annual total <10 mm), and stable snow cover is rare. Surface snow typically melts within 1–3 days, and snow depths exceeding 5 cm are uncommon. Temporary snow may accumulate in mountainous or elevated areas, but long-term snow cover is virtually absent in plain agricultural regions [22]. In Bachu County, the warmest months are July and August (summer), with a maximum temperature of 43 °C; January (winter) is the coldest month, with a minimum temperature of −25.1 °C. Snowfall is extremely scarce: the historical maximum snow depth is only 8 mm, precluding the formation of thick snow layers. Snow cover is unstable and short-lived, with persistent snow rare in most years; long-term snow cover is absent in both plain agricultural regions and desert areas of the county [22]. In Shawan, summer highs peak in July and August, with a monthly average high of 34 °C and a historical extreme of 42.9 °C. Winter lows are most severe in January and February: January averages −16 °C (extreme low: −23 °C), while February averages −18 °C (extreme low: −27 °C). Snowfall in Shawan is greater than in Maigaiti and Bachu, with thicker snow cover averaging about 12 cm and persisting until early April of the following year [22]. Other detailed information on climate, topography, and geographical coordinates of these three counties is provided in Huang and Wang [26].

### 2.2. Data of Climate and H. armigera

Meteorological parameters were recorded at weather stations operated by the meteorological administrations of Maigaiti, Bachu, and Shawan, located on the periphery of each county. Adult moths were trapped using a 20 W black light lamp (Jiaduo Technology, Industry and Trade Co., Ltd., Hebi, China), deployed in an open field at 1.5 m above the ground level with no trees or tall buildings within the immediate vicinity. The distance between each weather station and corresponding light trap was approximately 300 m. The lamp was activated at dusk and deactivated at dawn, with trapping periods differing by location: Shawan (early April–late September, 1996–2018), Bachu (early April–late October, 1991–2015), and Maigaiti (early April–late October, 1989–2017). A new lamp tube was replaced annually, and trapped adults were counted daily in accordance with national standards. Zero captures were recorded when no moths were collected but excluded from subsequent analyses. *H. armigera* moths were identified following standard protocols [27,28].

### 2.3. Statistical Methods

The interannual population change ratio was quantified using *R*-value, calculated as *R* = [Log_2_(N) + 1]/[Log_2_(N − 1) + 1]. Here, N denotes the number of moths in year N, and N − 1 denotes the number in year N − 1, *R* > 1 indicates population growth, while *R* < 1 indicates decline. For the comparing of monthly moth counts between consecutive years, the same formula was applied, with N representing the count in a specific month of the current year and N − 1 representing the count in the same month of the preceding year.

Annual temperature difference was defined as the difference between the current year’s and the previous year’s, encompassing three metrics: annual average temperature (*T*_mean_), annual average (*T*_max_), and annual minimum temperature (*T*_min_). All references to *T*_mean_, *T*_max_, and *T*_min_ in this study denote the annual average values.

Quarterly and monthly temperature differences were defined as the temperature of a specific quarter (or month) in the current year minus that of the same quarter (month) in the previous year, encompassing three temperature metrics: *T*_mean_, *T*_max_, and *T*_min_. All metrics represent the average values for the respective quarter (or month).

Linear regression was used to analyze trends in temperature versus trapped moth abundance, moth abundance versus growth days, and phenology over time. Pearson correlation analysis, regression functions, and Partial Least Squares (PLS) regression were applied to examine the relationship between the *R*-value and temperature difference—with PLS further used to mitigate multicollinearity and calculate the relative contribution of each temperature anomaly factor. All statistical analyses were performed using SPSS 26.0 for Windows (SPSS Inc., Chicago, IL, USA). PLS facilitates comparative analysis of multiple response and explanatory variables [29], demonstrates strong resistance to overfitting, and outperforms principal component analysis (PCA) in performance [30]. Statistical significance was defined as *p* < 0.05, and all graphs were generated using SigmaPlot 12.5 for Windows.

In this study, the four seasons were defined as follows: spring (March–May), summer (June–August), autumn (September–November), and winter (December–February).

## 3. Results

### 3.1. Population Changes of Size and R-Value

**Figure 1:**
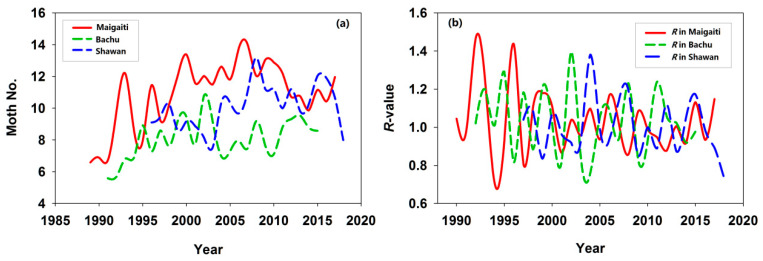
(**a**) Annual variation in trapped *H. armigera* moth abundance across three study regions. (**b**) Annual population change *R*-value. The moth number of *H. armigera* was expressed as Log_2_(moth number in a year) + 1.

*H. armigera* populations in Maigaiti, Bachu, and Shawan increased annually by 0.131, 0.079, and 0.085, respectively; only Shawan’s population growth was non-significant (Table 1). Maigaiti exhibited the fastest growth rate (Table 1), with population sizes peaking in 2007 (Maigaiti), 2002 (Bachu), and 2008 (Shawan) (Figure 1a). The Maigaiti population showed extreme peaks and troughs, while Bachu and Shawan populations displayed irregular fluctuations. Annual *R*-values fluctuated markedly but trended toward gradual narrowing (Figure 1b), indicating that post-peak population decline was accompanied by reduced interannual variability in population size. The proportions of *R* > 1 (indicating growth) were 53.57% (15/28) for Maigaiti, 58.33% (14/24) for Bachu, and 45.45% (10/22) for Shawan, with Shawan population showing an overall declining trend. Interannual *R*-value changes reflected responses to environmental variability: as shown in Figure 1, *H. armigera* populations were highly interannually volatile, a hallmark of r-selected species—this volatility enables rapid exploitation of favorable conditions (triggering outbreaks) and susceptibility to adverse factors (causing population crashes). For comparative purposes, population trend and *R*-value plots derived from untransformed observational data (no logarithmic transformation) are presented in Figure S1.

### 3.2. Temperature Affected Annual R-Value

#### 3.2.1. Changes of Maigaiti Population Annual *R*-Value

**Figure 2:**
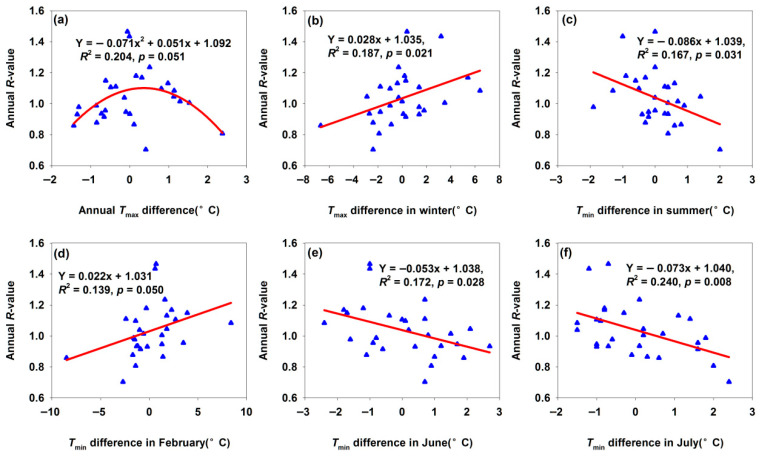
Temperature differences affected annual *R*-value for Maigaiti moth population. (**a**) Annual *T*_max_ difference; (**b**) *T*_max_ difference in winter; (**c**) *T*_min_ difference in summer; (**d**) *T*_min_ difference in February; (**e**) *T*_min_ difference in June; (**f**) *T*_min_ difference in July.

The annual *R*-value peaked when the annual *T*_max_ difference was approximately 0.5 °C (Figure 2a). This indicated that moderate annual *T*_max_ difference increases (<0.5 °C) promoted population growth by accelerating development, enhancing reproductive rates, and expanding the growing season. In contrast, an excessive interannual *T*_max_ difference (>0.5 °C) was observed to introduce heat stress, which appeared to compromise survival rates and reproductive performance—implying the putative existence of an optimal range of annual maximum temperature conducive to population proliferation. The annual *R*-value in Maigaiti increased significantly with a greater winter *T*_max_ difference (Figure 2b) and February *T*_min_ difference (Figure 2d). During winter, *H. armigera* exists as overwintering pupae; a larger temperature difference indicated faster warming rates. Warmer winters reduce cold-induced mortality of overwintering pupae of *H. armigera* [25,31,32], accelerate pupal development, and promote earlier, more synchronized adult emergence—extending the reproductive period [16]. Conversely, the annual *R*-value decreased with an increasing summer *T*_min_ difference (Figure 2c) and *T*_min_ difference in June and July (Figure 2e,f). June and July represent peak larval feeding and reproductive periods for *H. armigera*. Excessively high *T*_min_ during these months exerts multiple adverse effects: (a) increased larval metabolic costs (e.g., higher energy expenditure for thermoregulation), reducing nutrient storage for pupation and reproduction; (b) heat stress that lowers larval survival, pupal weight, and adult fecundity; (c) disrupted the synchronization between the pest’s life cycle and cotton’s susceptible growth stages (e.g., boll formation), decreasing resource utilization efficiency and inhibiting population growth. These results indicated that temperature differences in different seasonal phases exerted distinct regulatory effects on *H. armigera* populations in Maigaiti.

#### 3.2.2. Changes of Bachu Population Annual *R*-Value

**Figure 3:**
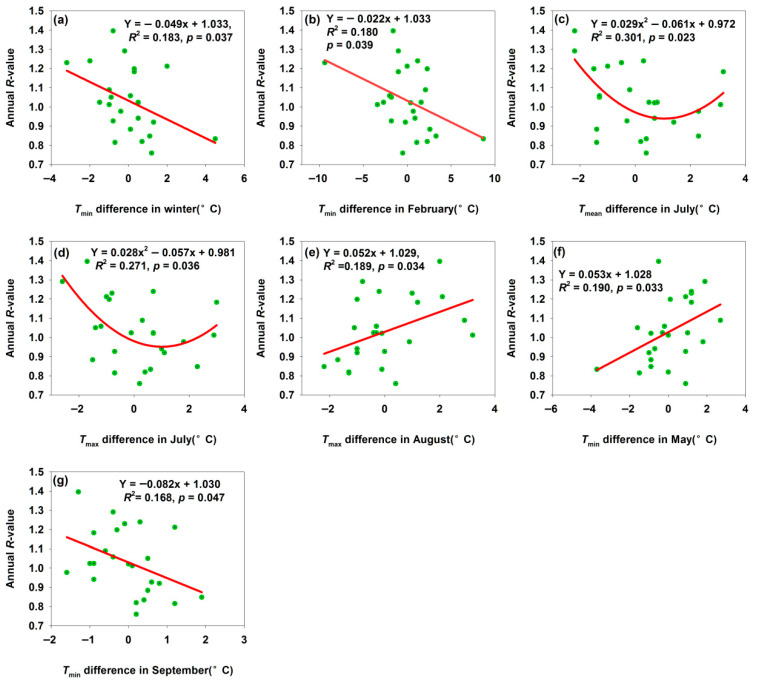
Temperature differences affected annual *R*-value for Bachu moth population. (**a**) *T*_min_ difference in winter; (**b**) *T*_min_ difference in February; (**c**) *T*_mean_ difference in July; (**d**) *T*_max_ difference in July; (**e**) *T*_max_ difference in August; (**f**) *T*_min_ difference in May; (**g**) *T*_min_ difference in September.

A 1 °C increase in *T*_min_ difference in winter and February decreased *R*-values by 0.049 and 0.022, respectively (Figure 3a,b). While warmer winters may initially appear beneficial, they can desynchronize *H. armigera*’s life cycle from host plant phenology (e.g., adults emerging before crops availability), reducing reproductive success. The annual *R*-value reached their minima when temperature differences in *T*_mean_ and *T*_max_ in July were about 1 °C (Figure 3c,d). July—the warmest month—coincides with *H. armigera*’s reproductive and larval feeding peak; temperatures ≥33 °C trigger summer diapause [33]. Moderate temperature deviations induced stress, excessive heat increased larval metabolic costs and mortality, while excessive cold slowed development. Extreme deviations might be even more detrimental, but the curve shape suggested there is an optimal stress range that most strongly suppressed population growth—reflecting *H. armigera*’s non-linear tolerance to temperature extremes during its key reproductive phase. A 1 °C temperature difference increase in *T*_max_ in August and *T*_min_ in May increased *R*-values by 0.052 and 0.053, respectively (Figure 3e,f). Warmer springs accelerated egg hatching and larval development, enabling the pest to exploit host plants earlier and boosting survival and reproduction. Warmer summers extended the growing season, supporting more generations or faster development and increasing annual offspring production—driving population growth. In contrast, a 1 °C increase in September *T*_min_ difference decreased the *R*-value (Figure 3g). September marks *H. armigera*’s final generation or pre-overwintering stage: cooling accelerates diapause initiation. An excessively early cold spell (large negative temperature difference) disrupts development of the final generation and reduces the overwintering quality; conversely, warmer temperatures extend feeding and reproduction, allowing larvae to continue developing instead of entering diapause. However, subsequent cooling causes these larvae to die from cold stress, failing to complete their life cycle and reducing the overwintering population base. These findings confirmed that temperature differences could be regarded as a key driver of *H. armigera* population dynamics, and the direction and shape of the relationship between temperature differences and *R*-value revealed how seasonal temperature variability fine-tunes population growth, underscoring the species’ sensitivity to climate and its role in pest outbreaks or declines.

#### 3.2.3. Changes of Shawan Population Annual *R*-Value

**Figure 4:**
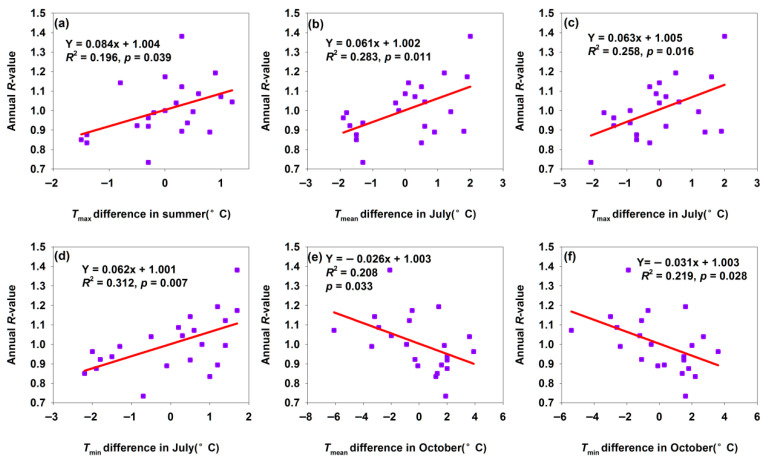
Temperature differences affected annual *R*-value for Shawan moth population. (**a**) *T*_max_ difference in summer; (**b**) *T*_mean_ difference in July; (**c**) *T*_max_ difference in July; (**d**) *T*_min_ difference in July; (**e**) *T*_min_ difference in October; (**f**) *T*_min_ difference in October.

In Shawan, increased temperature differences in summer, *T*_mean_, *T*_max_, and *T*_min_ of July, promoted *H. armigera* population growth (Figure 4a–d), while those in *T*_mean_ and *T*_min_ of October inhibited population growth (Figure 4e,f). Summer—particularly July—is critical for *H. armigera* reproduction and larval development: warmer temperatures accelerate metabolic rates, shorten developmental periods (e.g., faster egg hatching, larval molting, and pupation), and boost fecundity (more eggs laid by females) [1,34,35,36]. This supports additional generations or greater offspring production within a season, driving population growth. In October, *H. armigera* prepares for overwintering (as pupae). Warmer autumns disrupt the diapause induction: prolonged high temperatures prevent pupae from entering diapause properly, increasing their vulnerability to winter cold and reducing overwintering survival. Additionally, while warmer autumns may extend activity periods, host plants are typically harvested by October, leaving larvae/pupae with limited resources—lowering survival and thus inhibiting the following year’s population growth. A 1 °C increase in *T*_min_ difference and *T*_mean_ difference in October decreased *R*-values by 0.026 and 0.031, respectively (Figure 4e,f). This indicated that the inhibitory effect of the increase in *T*_min_ difference in October on *H. armigera* was greater than that of the *T*_mean_ difference in October. That is, increasing temperature in October inhibited population growth.

#### 3.2.4. Changes of Month Population *R*-Values in the Three Study Sites

The monthly *R*-value—calculated as the logarithm of the current month’s population size minus that of the same month in the preceding year—indicates population growth or decline.

**Figure 5:**
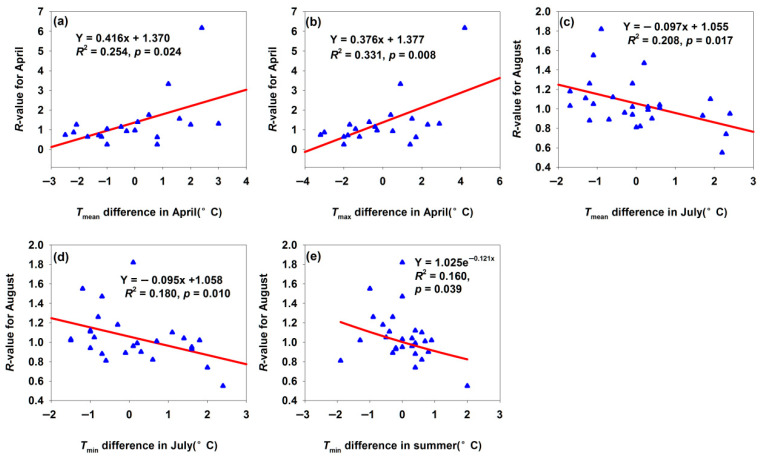
Temperature difference affected the *R*-values for month population in Maigaiti. (**a**) Relationship between *R*-value for April and *T*_mean_ difference in April; (**b**) relationship between *R*-value for April and *T*_max_ difference in April; (**c**) relationship between *R*-value for August and *T*_mean_ difference in July; (**d**) relationship between *R*-value for August and *T*_min_ difference in July; (**e**) relationship between *R*-value for August and *T*_min_ difference in summer.

For the Maigaiti *H. armigera* population, April marked the onset of spring activity. An increase of 1 °C in *T*_mean_ and *T*_max_ difference in April increased *R*-value by 0.416 and 0.376, respectively (Figure 5a,b). It suggested that warmer April temperatures accelerated the metabolic and development rates, facilitating early feeding and reproduction and thereby boosting population size. A 1 °C increase in *T*_mean_ and *T*_min_ difference in July increased *R*-value by 0.097 and 0.095, respectively (Figure 5c,d). July is a critical period for *H. armigera* reproduction and crop damage: mean temperature modulates reproductive capacity and development rate—excessively high temperature induces heat stress, inhibiting reproduction and development or increasing mortality, which reduces the August population. July minimum temperature influences night-time survival under heat stress and development: lower *T*_min_ alleviates daytime heat stress, benefiting night-time survival and development, while higher *T*_min_ exacerbates the heat stress, leading to a reduced August population. Notably, increased summer *T*_min_ difference decreased the August *R*-value (Figure 5e). As summer was the peak period of *H. armigera* activity, *T*_min_ affected the overall summer population dynamics: sustained high minimum temperatures might induce continuous heat stress, impairing feeding, development, and reproduction throughout the summer and reducing the late-season population base.

**Figure 6:**
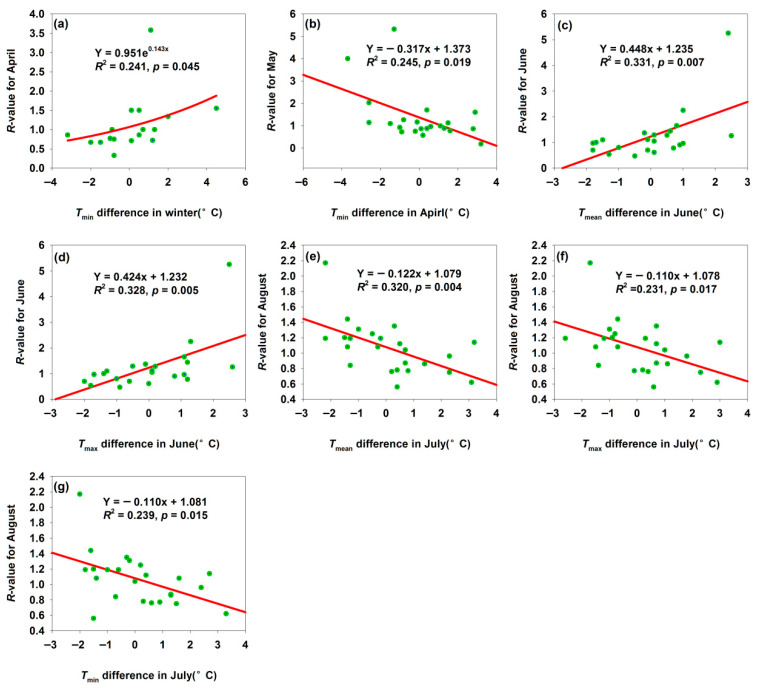
Temperature difference affected the *R*-values for month population in Bachu. (**a**) Relationship between *R*-value for April and *T*_min_ difference in winter; (**b**) relationship between *R*-value for May and *T*_min_ difference in April; (**c**) relationship between *R*-value for June and *T*_mean_ difference in June; (**d**) relationship between *R*-value for June and *T*_max_ difference in June; (**e**) relationship between *R*-value for August and *T*_mean_ difference in July; (**f**) relationship between *R*-value for August and *T*_max_ difference in July; (**g**) relationship between *R*-value for August and *T*_min_ difference in July.

For the Bachu *H. armigera* population, increased temperature differences in *T*_min_ in winter, *T*_mean_ in June, and *T*_min_ in July elevated the *R*-values (Figure 6a,c,d). Bachu mean winter *T*_min_ was −9.0 °C, with temperature difference ranging from −3.5 °C to 4.5 °C (Figure 6a); reduced low-temperature stress thus increased the overwintering survival rate. June was a core period for *H. armigera* reproduction and crop damage: suitable temperatures (*T*_mean_ = 24.9 °C, *T*_min_ = 32.5 °C, and small temperature differences of −2 °C to 2.6 °C) could accelerate egg hatching and larval development, shorten the generation cycle, and enable rapid population expansion—intensifying pest occurrence (Figure 6c,d). In contrast, increased temperature differences in *T*_min_ in April and *T*_mean_, *T*_max_, and *T*_min_ in July decreased the *R*-values (Figure 6b,e–g). April was a critical period for spring activity and reproduction: excessively high April *T*_min_ (large positive *T*_min_ differences) might disrupt *H. armigera* adaptation to spring temperature gradients (e.g., mismatched development rhythm with host plant phenology) or reduced egg/larval survival via temperature fluctuations or heat stress—lowering May population density and developmental progress. This reflected the disruptive effect of abnormal spring temperatures spring warming on the pest short-term development (Figure 6b). Excessively high July temperatures (large *T*_max_ differences) or night-time temperatures (large *T*_min_ differences) induce heat stress in *H. armigera*: (1) temperatures exceed the optimal development threshold, inhibiting enzyme activity, causing metabolic disorders, and reducing larval survival; (2) sustained high temperatures impair the mating and oviposition, lowering reproductive efficiency [37]. Thus, extreme July warming weakened the pest’s biological performance in August, demonstrating the inhibitory effect of summer temperature extremes (Figure 6e–g).

**Figure 7:**
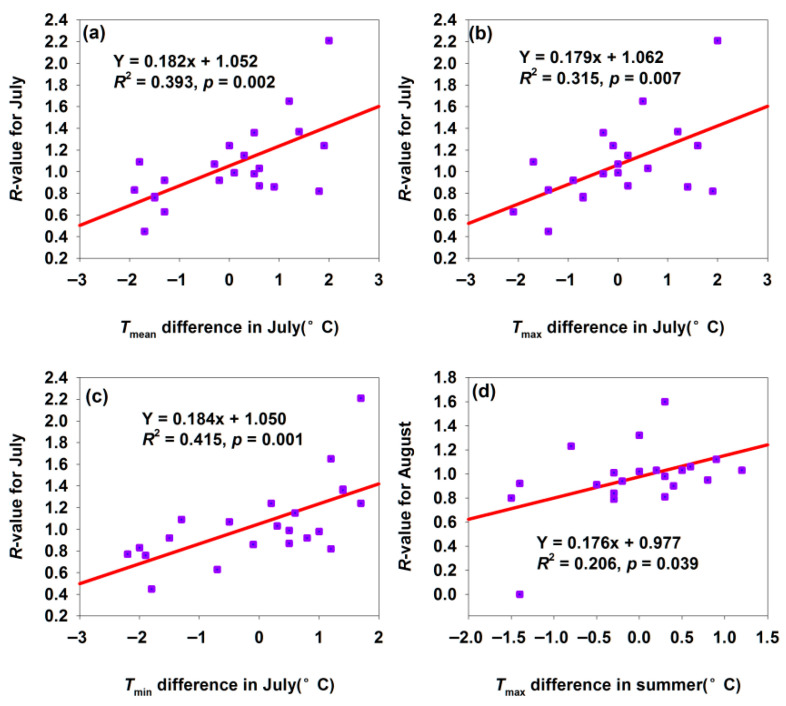
Temperature difference affected the *R*-values for month population in Shawan. (**a**) Relationship between *R*-value for July and *T*_mean_ difference in July; (**b**) relationship between *R*-value for July and *T*_max_ difference in July; (**c**) relationship between *R*-value for July and *T*_min_ difference in July; (**d**) relationship between *R*-value for August and *T*_max_ difference in summer.

For the Shawan *H. armigera* population, a 1 °C increase in temperature differences in *T*_mean_, *T*_max_, and *T*_min_ in July significantly elevated the July *R*-value by 0.182, 0.179, and 0.184, respectively (Figure 7a–c). July was critical for *H. armigera* growth, development, and reproduction. Increased suitable *T*_mean_ (positive differences) accelerated the development and enhanced adult fecundity (Figure 7a), with the *R*-value rising significantly with July warming *T*_mean_, reflecting the promotional effect of optimal July *T*_mean_ on development and population proliferation. Moderate *T*_max_ (below heat stress threshold) boosted metabolic activity, promoted larval feeding (accumulating nutrients for development/reproduction), and accelerated adult gonadal development. This improved July population density and reproductive efficiency, demonstrating the beneficial effect of moderate July *T*_max_ on the short-term biological performance (Figure 7b). Higher night-time *T*_min_ (positive differences) reduced energy consumption from cold stress, allowing *H. armigera* to allocate more resources to growth, development, and reproduction. Thus, the July *R*-value increased with night-time warming, highlighting the positive impact of July *T*_min_ on the energy allocation and biological performance (Figure 7c). Additionally, suitable summer *T*_max_ increases created conditions for the multi-generation development: early generations thrived under optimal *T*_max_, expanding the population base. This improved the August population size and development progress, reflecting summer warming’s role in promoting cross-month population persistence and development (Figure 7d).

#### 3.2.5. Changes of Population *R*-Values for May/September in the Three Study Sites

**Figure 8:**
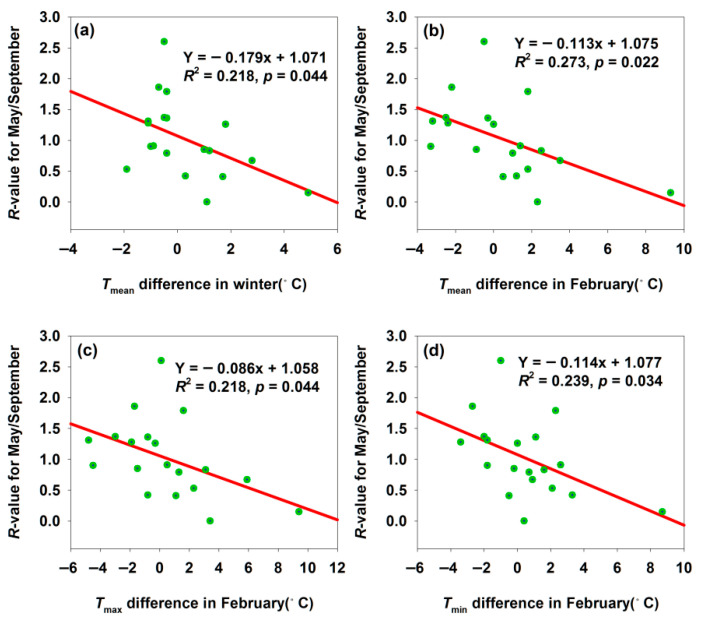
Temperature difference affected the *R*-values for May/September month population in Bachu. (**a**) Relationship between *R*-value for May/September and *T*_mean_ difference in winter; (**b**) relationship between *R*-value for May/September and *T*_mean_ difference in February; (**c**) relationship between *R*-value for May/September and *T*_max_ difference in February; (**d**) relationship between *R*-value for May/September and *T*_min_ difference in February.

Notably, winter temperature differences also affect *H. armigera* populations in the subsequent year. Among the three regions, only Bachu’s population showed a significant correlation (Figure 8), while Maigaiti and Shawan exhibited the same trends but non-significant correlations (*p* > 0.05). To quantify winter temperature difference-induced population changes, we calculated an *R*-value as *R* = [Log_2_(No. in May this year) + 1]/[Log_2_(No. in September last year) + 1]. A decreasing *R*-value with increasing temperature differences indicated high sensitivity of *H. armigera* to temperature variations. *H. armigera* overwinters in diapause: warmer winters disrupted diapause rhythms, increasing overwintering mortality, or accelerated metabolism (slowed under low temperatures), leading to insufficient energy reserves. The difference in adult abundance between September and the following May is the combined result of mortality in all ontogenetic stages (egg, larva, and pupa) and the reproductive output of autumn adults. The overwintering pupal survival rate is a critical but not the only factor driving the interannual variation in spring adult populations. Both scenarios reduce the spring population base (Figure 8a–d). The significant negative correlations suggested that *H. armigera* lacked sufficient flexibility to adapt to temperature fluctuations—deviations from optimal temperatures destabilized population and phenological dynamics. This reflected the species’ vulnerability: minor temperature changes disrupted normal biological processes, underscoring its susceptibility to climatic variability.

#### 3.2.6. Changes of Population *R*-Values for Summer in the Three Study Sites

**Figure 9:**
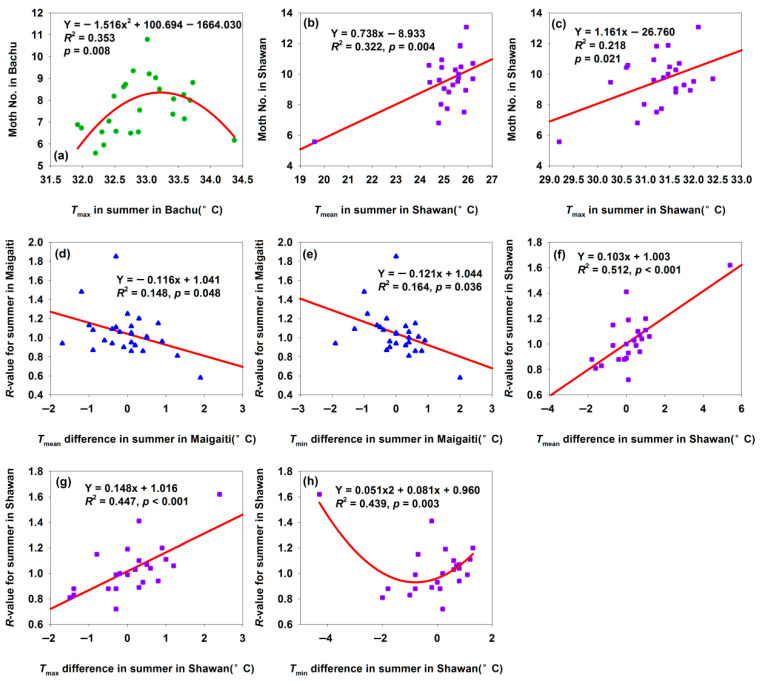
Relationships among moth number, *R*-value, and summer temperature difference. (**a**) Relationship between moth number and *T*_max_ in summer in Bachu; (**b**) relationship between moth number and *T*_mean_ in summer in Shawan; (**c**) relationship between moth number and *T*_max_ in summer in Shawan; (**d**) relationship between *R*-value for summer and *T*_mean_ in summer in Maigaiti; (**e**) relationship between *R*-value for summer and *T*_min_ in summer in Maigaiti; (**f**) relationship between *R*-value for summer and *T*_mean_ in summer in Shawan; (**g**) relationship between *R*-value for summer and *T*_max_ in summer in Shawan; (**h**) relationship between *R*-value for summer and *T*_min_ in summer in Shawan. * Moth number in (**a**–**c**) was expressed as “Log_2_(moth number in summer) + 1”.

The Bachu population began to decline when summer *T*_max_ reached 33 °C (Figure 9a), as this temperature triggered summer diapause [33]. In contrast, the Shawan population increased with rising *T*_max_ and *T*_mean_ (Figure 9b,c) since neither the *T*_max_ nor the *T*_mean_ exceeded 33 °C. Maigaiti’s *R*-values decreased with increasing *T*_mean_ and *T*_min_ differences (Figure 9d,e). Similar to Bachu (Figure 9a), Maigaiti’s population grew before the temperature reached 33 °C but declined after the temperature hit 33 °C—though this correlation was non-significant. Given that the temperature difference indicated warming trends and Maigaiti’s summer temperatures were higher than Bachu’s, the decreasing *R*-value with increasing temperature differences aligns with the 33 °C diapause threshold. Shawan’s summer temperatures never reached 33 °C, so no summer diapause occurred—explaining why its population increased with rising summer *T*_mean_ and *T*_max_ differences (Figure 9f,g). However, Shawan’s *R*-value first decreased then increased with *T*_min_ difference (Figure 9h); excluding the leftmost outlier, the *R*-value showed a positive trend with *T*_min_ difference. These results showed that moderate warming promoted *H. armigera* population growth, while temperatures exceeding 33 °C inhibited growth via summer diapause. Summer temperature variability was thus a key driver of population dynamics, with region-specific critical factors: (1) Maigaiti: summer *T*_mean_ and *T*_max_ differences; (2) Bachu: summer *T*_max_ difference; (3) Shawan: summer *T*_mean_, *T*_max_, and *T*_min_ differences.

### 3.3. Contribution Rates of Temperature Difference Factors

For the annual *R*-value of Maigaiti population, only two factors explained the observed population dynamics: winter *T*_max_ difference was the absolute dominant factor, contributing as high as 98.0% (Table 2). In contrast, summer *T*_min_ difference contributed a mere 2.0% (Table 2), exerting an extremely weak impact. These results indicated that the annual population changes of *H. armigera* in Maigaiti were almost entirely determined by winter *T*_max_ difference, with the effects of other temperature difference factors being negligible.

For the annual *R*-value of Bachu population, five factors jointly explained the observed population dynamics, with the top three factors accounting for 99.4% of the cumulative contribution (explained variance) (Table 2). The primary factor was May *T*_min_ difference (80.7%), followed by November *T*_max_ difference (15.8%) and August *T*_max_ difference (2.9%). Winter *T*_min_ difference (0.5%) and February *T*_min_ difference (0.1%) exerted negligible impacts. These results indicated that the *H. armigera* population in Bachu was jointly affected by temperature differences in across spring, summer, and autumn, with the spring (May) *T*_min_ difference as the core driver.

As to the annual *R*-value of Shawan population, only two factors fully explained the observed population dynamics: July *T*_min_ difference (99.4%), the dominant factor, and July *T*_mean_ difference (0.6%), which exerted an extremely weak influence (Table 2). These results indicated that annual *H. armigera* population changes in Shawan were almost entirely determined by July *T*_min_ difference, while the effects of other temperature difference factors could be negligible.

Thus, the *R*-value prediction models for the moth population in Maigaiti, Bahcu, and Shawan were Y = 0.002X_1_ − 0.006X_2_ − 1.065, adjusted *R*^2^ = 100%; Y = 0.007X_1_ − 0.006X_2_ + 0.009X_3_ − 0.005X_4_ − 0.002X_5_ + 0.820, adjusted *R*^2^ = 100%; and Y = 0.002X_1_ + 0.001X_2_ + 0.963, adjusted *R*^2^ = 100%, respectively. The X in the equation corresponded to the factors in Table 2 respectively. These models enable prediction of *H. armigera* population changes using the respective factors in Table 2, with such changes reflecting the species’ vulnerability to temperature variability.

### 3.4. Impacts of Phenology of Moth on Population Sizes in Three Regions

**Figure 10:**
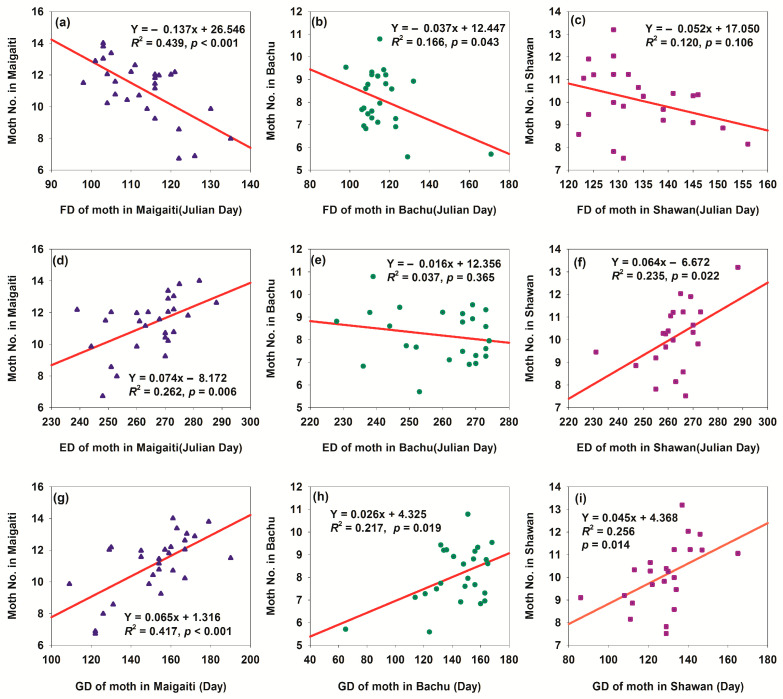
Relationships between phenology and moth number of *H. armigera* in three regions. (**a**) Relationship between FD of moth in Maigaiti and moth number; (**b**) relationship between FD of moth in Bachu and moth number; (**c**) relationship between FD of moth in Shawan and moth number; (**d**) relationship between ED of moth in Maigaiti and moth number; (**e**) relationship between ED of moth in Bachu and moth number; (**f**) relationship between ED of moth in Shawan and moth number; (**g**) relationship between GD of moth in Maigaiti and moth number; (**h**) relationship between GD of moth in Bachu and moth number; (**i**) relationship between GD of moth in Shawan and moth number. The moth number of *H. armigera* was expressed as Log_2_(moth number in a year) + 1.

Climate warming changed the phenology of *H. armigera* moth across the three regions, including first appearance date (FD), end disappearance date (ED), and growth duration (GD) of adult moths (Figure S2). Specifically, FD was advanced (Figure S2a–c), ED was delayed (Figure S2e,f), and GD was prolonged (Figure S2g–i). Advanced FD accelerated *H. armigera* growth and development (Figure 10a–c), while delayed ED extended its growth period (Figure 10d–f). Collectively, these shifts prolonged the total growth cycle (Figure 10g–i), leading to an increased population size (Figure 10)—indicating that warmer temperatures favor *H. armigera* population expansion. Linear relationship parameters varied among the three regions, reflecting regional differences in the species’ phenological response to warming. These differences might stem from variations in local climatic conditions (e.g., temperature and precipitation patterns), geographical environments (e.g., altitude and terrain), and ecosystem characteristics. Relationships between phenological traits (FD, ED, and GD) and the population size, combined with inter-regional phenological differences, highlight limitations in *H. armigera*’s phenological adaptability to temperature-driven environmental changes—underscoring its vulnerability to climate variability.

## 4. Discussion

This study quantified the fluctuations in *H. armigera* population size at multiple time scales (monthly, seasonal, and annual) in response to temperature differences. Phenological shifts driven by climate change also contribute to population variability. Furthermore, our findings indicated that geographically distinct populations were regulated by unique meteorological factors and regulatory patterns—highlighting the need for differentiated pest management strategies. These results provided a reference for investigating the responses of other pest species to climate change.

Overwintering pupae of *H. armigera* served as the population “seed bank” for the following year. Winter temperature changes affected overwintering pupae survival, thereby influencing the following year’s population size. Increased temperatures raise insects’ metabolic rates, enhance energy consumption, and affect their overwintering survival [2,38], leading to higher mortality. However, in this study, increasing winter temperature differences were associated with distinct population responses across regions: Bachu, a significant downward trend (Figure 3a); Maigaiti, a significant upward trend (Figure 2b); Shawan, a non-significant slight upward trend (Y = 0.007x + 1.004, *R*^2^ = 0.007, *p* = 0.721). Similarly, increased February *T*_min_ differences induced the following: Maigaiti, a significant population growth (Figure 2d); Bachu, a significant population decline (Figure 3b); Shawan, a non-significant slight population decline (Y = −0.004x + 1.005, *R*^2^ = 0.012, *p* = 0.624). These results indicated that a winter temperature increase had a significant impact on the Maigaiti and Bachu populations but had negligible effects on the Shawan population—reflecting divergent responses of geographical distinct populations to winter temperature variability. This regional variation might be related to soil properties and winter snow cover thickness, as snow cover and soil exert a buffering effect on microclimate of overwintering pupae [31], with snow cover thicknesses significantly impacting *H. armigera* overwintering mortality [32]. The average winter snow cover thickness in Maigaiti, Bachu, and Shawan was 0.39 cm, 0.20 cm, and 13.3 cm, respectively. Consequently, populations in Maigaiti and Bachu—with minimal snow cover—were more sensitive to temperature fluctuations, exhibiting distinct vulnerabilities to winter warming/cooling.

Summer was the peak period for *H. armigera* reproduction and development, with population size during this season determining the annual population scale. Increased summer temperatures exerted distinct impacts on the *H. armigera* populations across the three regions. The Maigaiti population showed declined trends (Figure 2c,e,f and Figure 9d,e), and the Bachu population displayed decreased first then increased trends (Figure 3c,d) or a directly increased trend (Figure 3e), and the Shawan population exerted increased trends (Figure 4a–d and Figure 9f,g). The Maigaiti population response differed from the other two regions, as high temperatures directly impaired *H. armigera* growth, development, and reproduction (Figure 9a). The species exhibits optimal fecundity at 25 °C, while newly hatched larvae die at an average temperature of 35 °C [39]. No effective oviposition occurs at 33 °C [37], and a sustained 35 °C mainly disrupts adult oviposition behavior [16]. Notably, *H. armigera* enters summer diapause at 33 °C [33] but terminates diapause immediately when the temperature drops to 30 °C, resuming development [40]. These temperature-dependent physiological responses drive summer population fluctuations in the species. These results indicated that *H. armigera* were sensitive to elevated summer night-time temperatures, which might affect their growth, development, or reproduction. They lacked effective adaptations to cope with rising summer minimum temperatures, making them vulnerable in this season.

Table 2 revealed that seasonal characteristics of temperature difference factors might reflect inter-regional differences in *H. armigera*’s ecological adaptation. The Maigaiti population was dominated by winter *T*_max_ difference—likely because local population size was shaped by overwintering survival, with winter *T*_max_ difference directly determining overwintering success (Figure 2b). The Bachu population was influenced by May *T*_min_ difference, August *T*_max_ difference, and November *T*_max_ difference. May was the spring emergence period of *H. armigera*, August was their summer reproduction period, and November was the autumn preparation period for overwintering (Figure 3). This indicated that the Bachu *H. armigera* population was restricted by temperature differences during multiple key growth and development periods throughout the year, showing greater ecological niche sensitivity. The Shawan population was dominated by July *T*_min_ difference (Figure 4d). As July marked the peak of summer reproduction, July *T*_min_ difference affected the larval development rate or adult fecundity, directly determining the annual population size. These findings suggested that geographically distinct *H. armigera* populations were regulated by unique temperature drivers, with varying patterns of control over annual population dynamics. This implies divergent responses to climate change and differing degrees of vulnerability among regional populations.

Although *H. armigera* populations showed increasing trends across the three regions, the magnitude of interannual fluctuations decreased. This phenomenon could be attributed to two main factors: (1) expansion of the population base; (2) *H. armigera* to a warming environment. Moderate warming may enhance the species’ adaptability, whereas high temperatures negatively impact its growth, development, and reproduction [1]. Winter and summer were critical time windows for *H. armigera*. The vulnerability of *H. armigera* populations to interannual abundance variations essentially reflected the imbalance between the abnormal temperature difference intensity during critical time windows and the population’s intrinsic tolerance. In low-vulnerability years, the temperature difference did not exceed threshold level (e.g., only short-term temperature differences occurred during the overwintering, while the reproductive period remained stable), allowing populations to compensate for losses through multi-generational reproduction. In contrast, high-vulnerability years were characterized by concurrent abnormal temperature differences in both overwintering and reproductive periods, which caused comprehensive damage from the initial population base to per-generation growth, ultimately leading to significant inter-annual fluctuations in population abundance.

Phenological shifts prolonged the annual growth period of *H. armigera*, thereby increasing its population size (Figure 10 and Figures S2)—a trend that would intensify with climate warming. However, since *H. armigera* enters summer diapause at 33 °C [33], its population is unlikely to increase significantly in summer. Instead, development would be delayed, leading to a larger autumn population. This not only would expand the overwintering base but also elevate the annual population size, promoting growth in the following spring and further boosting populations in the subsequent year. Such shifts might induce genetic alterations, which in turn would reduce the vulnerability of *H. armigera* to climate change and enhance its adaptability.

Collectively, the advanced FD, delayed ED, and extended annual GD did not act in isolation—their synergistic amplification not only boosted the current annual population size of *H. armigera* but also reshaped the pest’s ecological niche in agricultural systems. For instance, the additional one generation (3–4 generations/year) would expand the spatial range of damage (e.g., colonizing late-maturing crop varieties that were previously less affected) and intensify the pressure on crop protection during both early and late growing seasons.

From a long-term perspective, this study provides critical insights into the future impacts of *H. armigera* under climate change: as global warming continues to prolong suitable temperature windows for the pest (consistent with the prolonged summer suitable temperature ranges observed across the three regions), the aforementioned phenological shifts are likely to become more pronounced. This could lead to (1) a further increase in annual generations (potentially reaching 4–5 generations in some warm regions), (2) an earlier onset and later termination of the pest’s damage period, overlapping more extensively with the key growth stages of major crops (e.g., cotton boll formation and corn silking), and (3) a broader regional spread of *H. armigera* to areas that were previously too cold for its overwintering or reproduction. Such projections highlight the need for adaptive pest management strategies—e.g., adjusting the timing of pesticide application or planting pest-resistant crop varieties—to mitigate the escalating threat of *H. armigera* in a changing climate.

## 5. Conclusions

*H. armigera* populations were increasing across the three regions, while the magnitude of interannual fluctuations was decreasing. The main meteorological factors influencing interannual population changes differ by region: winter *T*_max_ difference (Maigaiti), May *T*_min_ difference (Bachu), and July *T*_min_ difference (Shawan). As the main meteorological factors (predominantly temperature) in these three regions continue to warm, the annual population size of *H. armigera* is projected to increase. Quantitatively, a 1 °C rise in *T*_mean_ in winter and February reduced spring population sizes by 17.9% and 11.3%, respectively. For annual populations, a 1 °C winter warming significantly decreased the Bachu population by 5.6%, significantly increased the Maigaiti population by 3.0%, and leads to a non-significant 0.7% increase in Shawan populations. Summer temperatures exceeding 33 °C suppress population growth. Advanced FD, delayed ED, and extended annual GD had collectively expanded the *H. armigera* population. These regional differences reflected divergent responses to climate change and varying degrees of vulnerability, with distinct regulatory patterns. Maigaiti was dominated by winter *T*_max_ difference. Bachu was constrained by temperature differences across multiple key growth and development stages annually, showing greater niche sensitivity. Shawan was dominated by July *T*_min_ difference.

## Figures and Tables

**Table 1:** Temporal trends of *H. armigera* population dynamics in Maigaiti, Bachu, and Shawan.

Population Site	Function	*R* ^2^	*p*
Maigaiti	Y = 0.131x − 250.823	0.294	0.002
Bachu	Y = 0.079x − 150.670	0.215	0.020
Shawan	Y = 0.085x − 160.845	0.168	0.052

**Table 2:** Contribution rates of temperature difference factors to annual *R*-values for *H. armigera* population sizes in three regions.

	Maigaiti Population	Bachu Population	Shawan Population
Factor 1	*T*_max_ difference in winter (98.0%)	*T*_min_ difference in May (80.7%)	*T*_min_ difference in July (99.4%)
Factor 2	*T*_min_ difference in summer (2.0%)	*T*_max_ difference in November (15.8%)	*T*_mean_ difference in July (0.6%)
Factor 3	/	*T*_max_ difference in August (2.9%)	/
Factor 4	/	*T*_min_ difference in winter (0.5%)	/
Factor 5	/	*T*_min_ difference in February (0.1%)	/
Adjusted *R*^2^	100%	100%	100%

* Note: The values in parentheses represent the contribution rate of this factor.

## Data Availability

The data presented in this study are available in the article and Supplementary Materials.

## Supplementary Materials

The following supporting information can be downloaded at https://www.mdpi.com/article/10.3390/insects17010040/s1, Table S1. *R*-value and temperature differences in Shawan. Table S2. *R*-value and temperature differences in Bachu. Table S3. *R*-value and temperature differences in Maigaiti. Table S4. Phenology and logarithm of adult abundance. Figure S1. Changes of moth number and *R*-value based on observation data. Figure S2. Phenology changes over time.
