# Supplementary material for: Temperature Changes Affect the Vulnerability of Cotton Bollworms, Helicoverpa armigera (Hübner)
Source: Insects. 2025 Dec 28;17(1):40. doi: 10.3390/insects17010040 (PMC12842468; doi:10.3390/insects17010040)
Supplement: Supplementary file 1 [file insects-17-00040-s001.zip › Table S1.R-value and temperature diffenrec in Shawan.pdf]

Year	Annual R for Shawan population	R for July in Shawan	R for August in Shawan	Tmax difference in summer in Shawan	Tmean difference in July in Shawan
1997	1.0396	1.0700	1.0300	0.2	-0.3
1998	1.0867	1.2400	1.0600	0.6	0
1999	0.8346	0.9800	0.0000	-1.4	0.5
2000	1.0723	1.1500		1	0.3
2001	0.9630	0.8300	1.0100	-0.3	-1.9
2002	0.9199	0.8700	0.8400	-0.3	0.6
2003	0.9227	0.4500	0.9100	-0.5	-1.7
2004	1.3816	2.2100	1.6000	0.3	2
2005	0.9942	1.3700	1.0300	0.5	1.4
2006	0.9371	0.9200	0.9000	0.4	-1.3
2007	1.1426	0.9900	1.2300	-0.8	0.1
2008	1.1935	1.6500	1.1200	0.9	1.2
2009	0.8508	0.7700	0.8000	-1.5	-1.5
2010	1.0000	0.9200	1.0200	0	-0.2
2011	0.8896	0.8600	0.9500	0.8	0.9
2012	1.1221	1.3600	0.9800	0.3	0.5
2013	0.8760	0.7600	0.9200	-1.4	-1.5
2014	1.0448	1.0300	1.0300	1.2	0.6
2015	1.1735	1.2400	1.3200	0	1.9
2016	0.9892	1.0900	0.9400	-0.2	-1.8
2017	0.8942	0.8200	0.8100	0.3	1.8
2018	0.7343	0.6300	0.7900	-0.3	-1.3

Tmax difference in July in Shawan	Tmin difference in July in Shawan	Tmean difference in October in Shawan	Tmin difference in October in Shawan
0	-0.5	3.6	2.7
-0.1	0.2	-2.9	-2.6
-0.3	1	1.2	2.2
0.2	0.6	-6.1	-5.4
-1.4	-2	3.9	3.6
0.2	0.5	2	1.5
-1.4	-1.8	-0.3	-1.1
2	1.7	-2.1	-1.9
1.2	1.4	1.8	2
-0.9	-1.5	2	1.5
0	0.5	-3.2	-3
0.5	1.2	1.4	1.6
-0.7	-2.2	1.3	1.4
-0.9	0.8	-0.9	-0.5
1.4	-0.1	-0.1	-0.1
-0.3	1.4	-0.7	-1.1
-0.7	-1.9	2	1.8
0.6	0.3	-2	-1.2
1.6	1.7	-0.5	-0.7
-1.7	-1.3	-3.4	-2.4
1.9	1.2	1.6	0.3
-2.1	-0.7	1.9	1.6
