# Supplementary material for: Temperature Changes Affect the Vulnerability of Cotton Bollworms, Helicoverpa armigera (Hübner)
Source: Insects. 2025 Dec 28;17(1):40. doi: 10.3390/insects17010040 (PMC12842468; doi:10.3390/insects17010040)
Supplement: Supplementary file 1 [file insects-17-00040-s001.zip › Table S2.R-value and temperature difference in Bachu.pdf]

Year	Annual R for Bachu population	R for May/September in Bachu	R for April in Bachu	R for May in Bachu	R for June in Bachu	R for August in Bachu
1992	1.0215	0.0000			0.4700	0.8700
1993	1.1982				2.2500	1.2000
1994	1.0117	1.2800		5.3200	0.9600	0.6200
1995	1.2909	2.6000		1.2600	1.3700	1.1900
1996	0.815	1.3600		0.7700	0.5400	1.4400
1997	1.1829			1.1200	1.6500	1.1400
1998	0.8837	0.9100	0.7100	0.8600	0.7000	1.0800
1999	1.2118	1.2600	1.3400	1.0900	1.4400	1.3100
2000	1.0239	1.8600	0.6700	0.8800	1.2900	0.7700
2001	0.8197	1.7900	1.0000	0.7500	0.7800	0.7600
2002	1.3959		0.3300	1.1600	1.0500	2.1700
2003	0.848	0.4200	3.5800	0.7200	0.8000	0.7500
2004	0.7596	0.4100	0.7200	0.8600	0.6100	0.7800
2005	1.0504	1.3100	1.0000	2.0300		0.8400
2006	1.089	0.5300	0.7700	0.5700		1.1900
2007	0.9409	0.6700	1.5000	1.6000	5.2500	1.0400
2008	1.2299		0.8600	1.1400	1.2800	1.2500
2009	0.8337	0.1500	1.5500	0.1800	1.0000	0.5600
2010	0.927	0.9000	0.7500	4.0000	0.7000	1.0800
2011	1.2391		0.6700	1.7000	1.2600	1.3500
2012	1.0579	1.3700	1.5000	0.9900	1.1000	1.1900
2013	1.0236	0.8300	0.8600	0.8700	0.9000	1.1200
2014	0.9203	0.8500	1.0000	0.9200	0.9700	0.8600
2015	0.9772	0.7900		0.9600	1.1100	0.9600

Tmean difference in winter in Bachu	Tmin difference in winter in Bachu	Tmean difference in February in Bachu	Tmax difference in February in Bachu	Tmin difference in February in Bachu	Tmin difference in April in Bachu
1.1	0.5	2.3	3.4	0.4	2.1
0.4	0.3	1.4	0.9	2.3	-1
-1.1	-1	-2.4	-1.9	-3.4	-1.3
-0.5	-0.2	-0.5	0.1	-1	-0.8
-0.4	-0.7	-0.3	-0.8	1.1	1.6
1.6	0.3	1.2	2.8	-1	1.5
-0.9	0.1	1.4	0.5	2.6	0.1
1.8	2	0	-0.3	0	-1.5
-0.7	-1.5	-2.2	-1.7	-2.7	1.3
-0.4	0.7	1.8	1.6	2.3	-0.2
-0.8	-0.8	-1.2	-0.6	-1.6	-0.1
0.3	1.1	1.2	-0.8	3.3	-0.9
1.7	1.2	0.5	1.1	-0.5	2.8
-1.1	-0.9	-3.2	-4.8	-1.8	-2.6
-1.9	-1	1.8	2.3	2.1	0.2
2.8	0.5	3.5	5.9	0.9	2.9
-4.5	-3.2	-10.9	-11.9	-9.4	-2.6
4.9	4.5	9.3	9.4	8.7	3.2
-1	-0.8	-3.3	-4.5	-1.8	-3.7
-1.7	-2	2.1	2.9	1.2	0.4
-0.5	0.1	-2.5	-3	-2	1.1
1.2	0.5	2.5	3.1	1.6	0.4
1	1.3	-0.9	-1.5	-0.2	-1
-0.4	-0.4	1	1.3	0.7	0.6

Tmin difference in May in Bachu	Tmean difference in June in Bachu	Tmax difference in June in Bachu	Tmean difference in July in Bachu	Tmax difference in July in Bachu	Tmin difference in July in Bachu
-0.9	-0.5	-0.9	0.7	0.7	1.3
0.1	1	1.3	-1.5	-0.9	-1.5
0	1	1.1	3.1	2.9	3.3
1.9	-0.2	-0.1	-2.2	-2.6	-1.8
-1.5	-1.3	-1.8	-1.4	-0.7	-1.6
1.2	0.8	1.1	3.2	3	2.7
-0.9	-0.1	-0.6	-1.4	-1.5	-1.4
0.9	0.6	1.2	-1	-1	-0.2
1	0.1	-0.5	0.8	-0.1	0.9
0	0.7	1.2	0.2	0.4	0.6
-0.5	0.1	0.1	-2.2	-1.7	-2
-0.9	-1	-1	2.3	2.3	1.5
0.9	0.1	0	0.4	0.2	0.3
-1.6	0.8	0.9	-1.3	-1.4	-0.7
2.7	-2.2	-2	-0.2	0.3	-0.6
-0.7	2.4	2.5	0.7	1	0
1.2	0.5	0.2	-0.5	-0.8	0.2
-3.7	-1.7	-1.4	0.4	0.6	-1.5
0.9	-1.8	-2	-0.3	-0.7	1.6
1.2	2.5	2.6	0.3	0.7	-0.3
-0.2	-1.5	-1.3	-1.3	-1.2	-1
-0.3	0.9	0.8	0.5	0.7	0.4
-1	-1.8	-1.7	1.4	1.1	1.3
1.8	-0.1	0.1	2.3	1.8	2.4

Tmax difference in August in Bachu	Tmin difference in September in Bachu
-0.1	0
-1	-0.3
3.2	0.1
-0.8	-0.4
-1.3	1.2
1.2	-0.9
-1.7	0.5
2.1	1.2
-0.4	-1
-1.3	0.2
2	-1.3
-2.2	1.9
0.4	0.2
-1.1	0.5
2.9	-0.6
-1	-0.9
1	-0.1
-0.1	0.4
0	0.6
-0.2	0.3
-0.3	-0.4
-0.3	-0.9
-1	0.8
0.9	-1.6
