# Supplementary material for: Temperature Changes Affect the Vulnerability of Cotton Bollworms, Helicoverpa armigera (Hübner)
Source: Insects. 2025 Dec 28;17(1):40. doi: 10.3390/insects17010040 (PMC12842468; doi:10.3390/insects17010040)
Supplement: Supplementary file 1 [file insects-17-00040-s001.zip › Table S3.R-value and temperature differences in Maigaiti.pdf]

Year	Annuarl R for Maigiati population	R for April in Maigaiti population	R for August in Maigaiti population	Annual Tmax difference in Maigaiti	Tmax difference in winter in Maigaiti
1990	1.0456			1.13	-2.9
1991	0.9782		0.8100	-1.31	1.4
1992	1.4651		1.4700	-0.06	0.4
1993	1.2353		1.8200	0.51	-0.3
1994	0.7036		0.5500	0.41	-2.4
1995	0.9312		1.1100	-1.35	1.4
1996	1.4348		1.5500	-0.01	3.2
1997	0.807		0.7400	2.38	-1.9
1998	1.1061	1.1500	1.1200	-0.49	1.4
1999	1.1791	0.8700	1.2600	0.16	0.2
2000	1.1104	1.2600	1.0400	-0.36	-1.8
2001	0.8655	1.0400	0.9000	0.11	-0.9
2002	1.0389	0.9300	1.0300	-0.14	-0.3
2003	0.9559	0.2500	0.9500	-0.63	1.8
2004	1.0974	6.1700	1.0500	0.81	-1
2005	0.9358	0.7200	0.9600	-0.73	-2.7
2006	1.1685	0.9700	1.2600	0.31	5.4
2007	1.0152	1.3100	1.0100	1.29	0
2008	0.8587	0.7400	0.8200	-1.44	-6.7
2009	1.084	1.5600	1.0200	1.14	6.4
2010	0.9877	0.6400	1.0200	-0.86	-1
2011	0.9472	0.6200	0.9400	-0.1	-1.8
2012	0.8779	1.3900	0.8900	-0.85	-2.4
2013	1.0056	1.7500	0.9900	1.53	3.5
2014	0.9155	0.6400	0.9300	-0.66	0.3
2015	1.1318	0.2500	1.1000	0.98	-0.4
2016	0.9337	3.3200	0.8800	0	0.1
2017	1.1488	1.2600	1.1800	-0.62	0.3

Tmin difference in summer in Maigaiti	Tmin difference in February in Maigaiti	Tmean difference in April in Maigaiti	Tmax difference in April in Maigaiti	Tmin difference in June in Maigaiti	Tmean difference in July in Maigaiti
1.4	1.8	0.7	0.6	2.1	0.8
-1.9	-1.5	-0.6	-0.4	-1.6	0
0	0.7	2.7	3.1	-1	0.2
0	1.6	-0.2	0.8	0.7	-0.9
2	-2.7	-2	-2.7	0.7	2.2
-0.4	-0.2	-1	-1.9	0.4	-1.3
-1	0.6	0.2	-0.3	-1	-1.1
0.4	-1.4	3.9	5.7	0.9	2.3
0.4	2.7	-0.5	-0.4	0	-0.6
-0.9	-0.3	-2.2	-3	-1.2	-1.2
0.3	-2.4	2	2.3	0.7	0.6
0.8	1.4	-1	-1.4	1	0.4
0	-1	-0.3	0.5	0.2	-1.7
0	3.5	-1	-2	-0.9	2.4
-0.5	-1.4	2.4	4.2	0.1	-1.1
0.3	-1.2	-1.3	-1.8	1.2	-0.3
-0.3	2.3	0	-0.3	-1.8	-0.1
0.7	-0.6	3	2.9	1.5	0.6
0.6	-8.5	-2.5	-3.2	1.9	0.1
-1.3	8.4	1.6	1.5	-2.4	0.3
0.9	-1.6	-1.7	-1.2	-0.8	-0.1
-0.2	1.3	0.8	1.7	1.7	-0.1
-0.3	-1.7	0.1	-0.7	-1.1	-0.7
0.4	1.3	0.5	0.4	0.8	0.3
-0.2	-0.9	-1.2	-2	-0.6	1.7
0.6	1.8	0.8	1.4	-0.4	1.9
0.4	-1.3	1.2	0.9	2.7	-1.2
-0.6	3.9	-2.1	-1.7	-1.7	-1.7

Tmin difference in July in Maigaiti
0.2
-0.6
-0.7
0.1
2.4
-1
-1.2
2
-1
-0.8
1.4
0.3
-1.5
1.6
-0.9
0.1
-0.8
0.7
0.6
-1.5
1.8
-1
-0.1
0.2
1.6
1.1
-0.7
-0.3
