# Supplementary material for: Temperature Changes Affect the Vulnerability of Cotton Bollworms, Helicoverpa armigera (Hübner)
Source: Insects. 2025 Dec 28;17(1):40. doi: 10.3390/insects17010040 (PMC12842468; doi:10.3390/insects17010040)
Supplement: Supplementary file 1 [file insects-17-00040-s001.zip › Table S4.Phenology and moth number.pdf]

Year	FD in Maigaiti	FD in Bachu	FD in Shawan	ED in Maigaiti	ED in Bachu	ED in Shawan	GD in Maigaiti	GD in Bachu	GD in Shawan	Moth number in Maigaiti
1989										6.58
1990	126			248			122			6.88
1991	122	129		244	253		122	124		6.73
1992	130	171		239	236		109	65		9.86
1993	121	108		251	268		130	160		12.18
1994	122	123		253	269		131	146		8.57
1995	135	132		261	273		126	141		7.98
1996	116	123	145	270	244	231	154	121	86	11.45
1997	116	108	124	271	273	258	155	165	134	9.24
1998	104	111	145	271	260	266	167	149	121	10.22
1999	104	111	122	271	247	255	167	136	133	12.05
2000	105	117	139	268	249	247	163	132	108	13.38
2001	106	107	151	251	239	263	145	132	112	11.58
2002	120	115	156	249	266	267	129	151	111	12.03
2003	98	114	131	288	270	260	190	156	129	11.5
2004	111	107	141	278	270	270	167	163	129	12.62
2005	116	111	146	275	274	259	159	163	113	11.81
2006	103	115	139	282	266	261	179	151	122	13.8
2007	103	109	123	264	238	288	161	129	165	14.01
2008	116	118	129	273	252	266	157	134	137	12.03
2009	103	106	132	271	262	273	168	156	141	13.04
2010	101	114	129	273	228	262	172	114	133	12.88
2011	110	118	129	270	273	262	160	155	133	12.2
2012	112	111	125	273	269	272	161	158	147	10.71
2013	106	98	131	260	266	259	154	168	128	10.77
2014	114	109	135	263	273	265	149	164	130	9.86
2015	116	121	129	270	269	269	154	148	140	11.16
2016	109		124	260		270	151		146	10.42
2017	117		134	262		255	145		121	11.97
2018			129			258			129	

Moth number in Bachu	Moth number in Shawan
5.58	
5.7	
6.83	
6.91	
8.92	
7.27	9.1
8.6	9.46
7.6	10.28
9.21	8.58
9.43	9.2
7.73	8.86
10.79	8.15
9.15	7.52
6.95	10.39
7.3	10.33
7.95	9.68
7.48	11.06
9.2	13.2
7.67	11.23
7.11	11.23
8.81	9.99
9.32	11.21
9.54	9.82
8.78	10.26
8.58	12.04
	11.91
	10.65
	7.82
