# Supplementary figures and images for: Temperature Changes Affect the Vulnerability of Cotton Bollworms, Helicoverpa armigera (Hübner)
Source: Insects. 2025 Dec 28;17(1):40. doi: 10.3390/insects17010040 (PMC12842468; doi:10.3390/insects17010040)

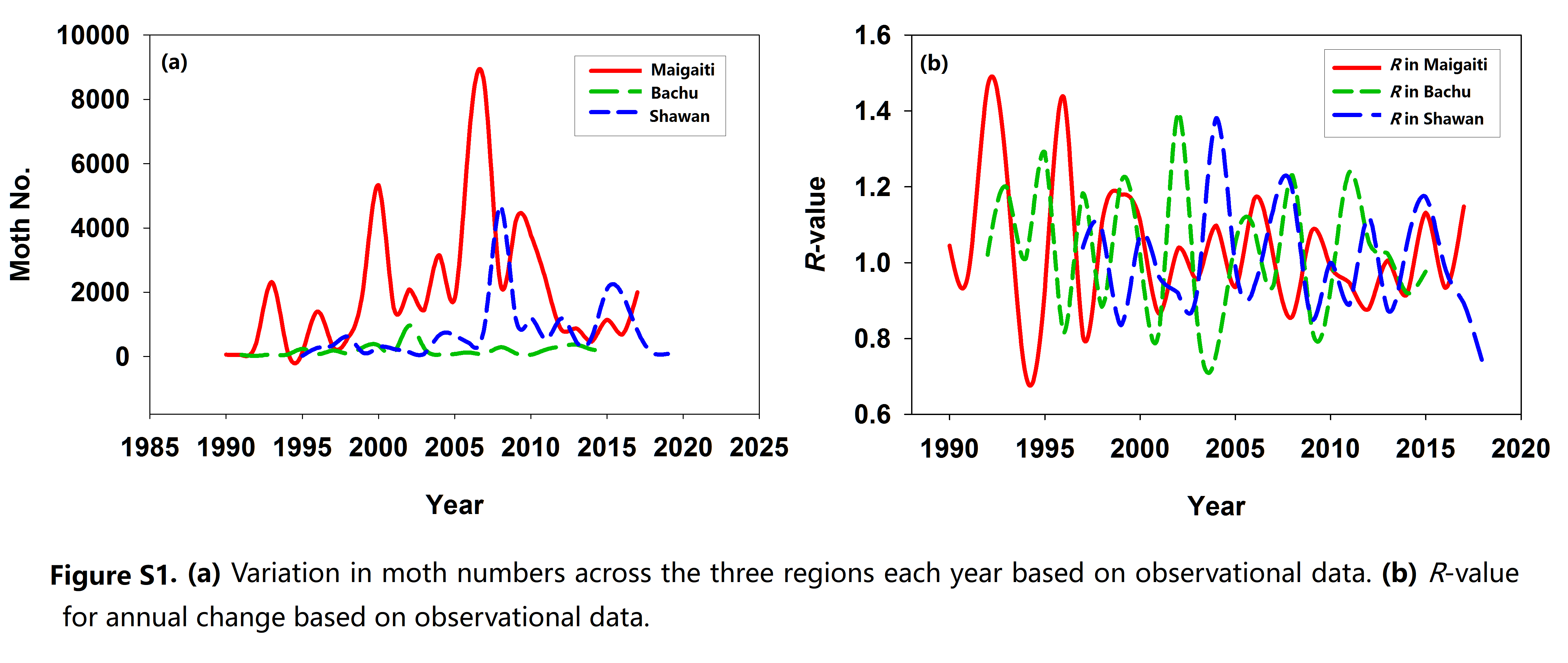

Supplement: Supplementary file 1 [file insects-17-00040-s001.zip › Figure S1.TIF]

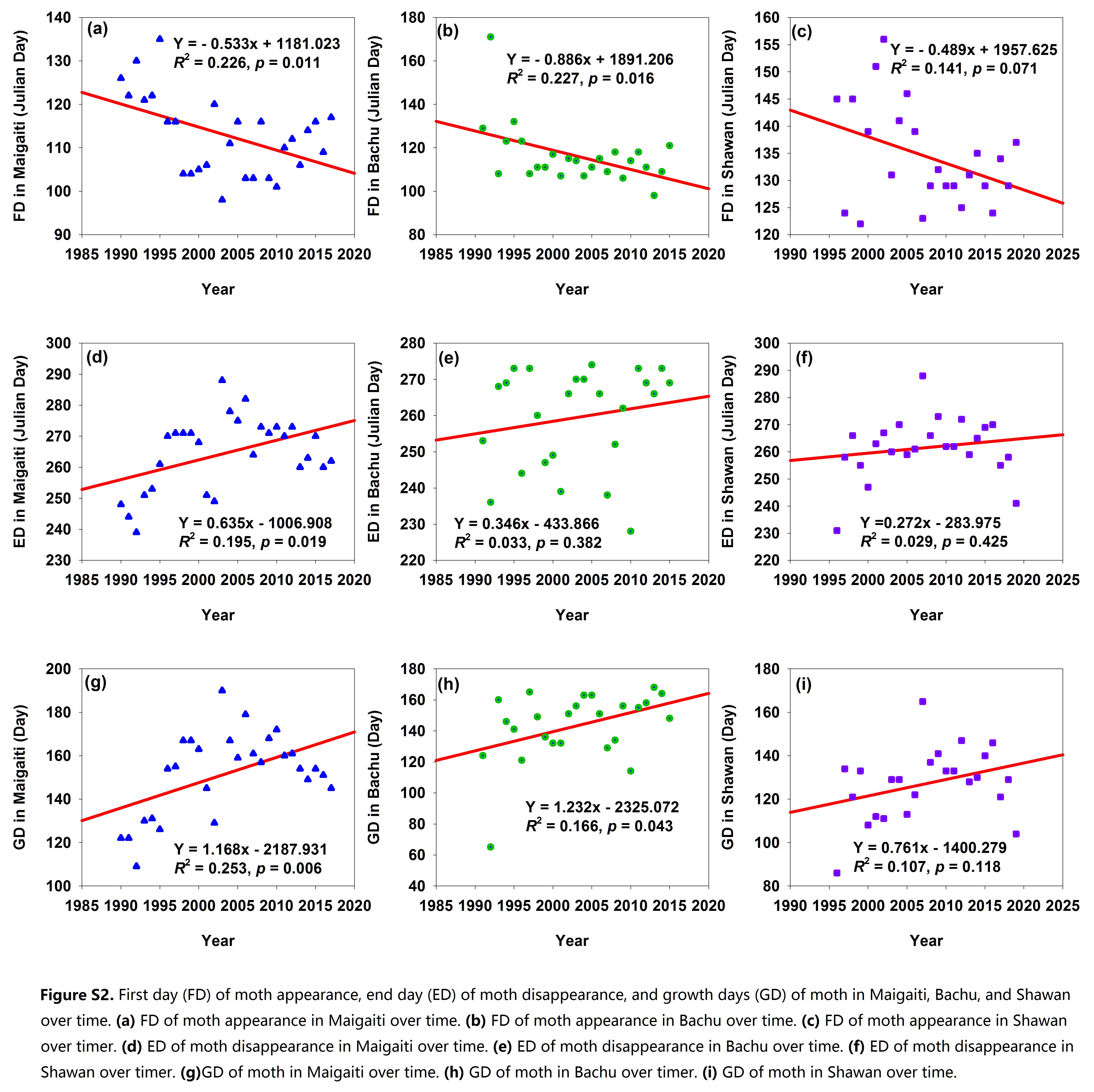

Supplement: Supplementary file 1 [file insects-17-00040-s001.zip › Figure S2.TIF]
